# Alterations in the Gut Microbiome of *Loxostege sticticalis* in Response to Temperature and Photoperiod Changes

**DOI:** 10.3390/insects17080802

**Published:** 2026-08-03

**Authors:** Jinghao Ji, Yanjun Xu, Yang Li, Hongxing Xu, Zhongxian Lu, Ronggen Wen, Sunan Shen, Yan Wu, Jianlei Huang, Jiawen Guo

**Affiliations:** 1 College of Agriculture and Forestry, Hebei North University, Zhangjiakou 075000, China; jijinghao2026@163.com (J.J.); XuYanJ@hebeinu.edu.cn (Y.X.); 2 State Key Laboratory for Quality and Safety of Agro-Products, Institute of Plant Protection and Microbiology, Zhejiang Academy of Agricultural Sciences, Hangzhou 310021, China; 13588332930@163.com (H.X.); luzxmh@163.com (Z.L.); rgw_ue@163.com (R.W.); s3234197322@163.com (S.S.); 3 Lishui Municipal General Station of Soil, Fertilizer, Plant Protection and Energy, Lishui 323000, China; lishuizb@163.com; 4 Guizhou Provincial Key Laboratory for Rare Animal and Economic Insect of the Mountainous Region, Department of Biology and Engineering of Environment, Guiyang University, Guiyang 550005, China; 15150535703@163.com

**Keywords:** *Loxostege sticticalis*, gut microbiome, temperature, photoperiod, adaptability

## Abstract

*Loxostege sticticalis* is a destructive pest that survives winter by entering diapause. Photoperiod and temperature trigger this dormancy, but their effects on gut bacteria are unclear. We hypothesized that photoperiod and temperature interact to alter the gut bacterial community structure and function and that these microbial changes may be involved in diapause preparation. To test this hypothesis, this study investigated how different photoperiod (L10:D14 and L16:D8) and temperature (18, 22, 26 and 30 °C) combinations shape the gut bacterial communities and their predicted functions in *L. sticticalis* larvae using 16S rRNA sequencing. The larval gut contained 29 phyla, 52 classes, 128 orders, 205 families, 407 genera, and 281 ASVs at species-level taxonomic annotation. Under a short-day photoperiod (L10:D14), the 26 °C treatment caused a dramatic loss of *α*-diversity and an almost complete dominance of *Enterococcus* (99.96%). *β*-diversity clearly separated this group from all others. LEfSe identified *Enterococcus* as its exclusive biomarker. Functionally, this group showed significantly elevated carbohydrate metabolism but reduced amino acid metabolism, energy metabolism, and metabolism of cofactors and vitamins. No such simplification occurred under long-day photoperiod (L16:D8) or at other temperatures under short-day conditions. These findings raise the hypothesis that gut microbiota may participate in metabolic reprogramming during diapause preparation and that environmental factors such as photoperiod and temperature could mediate host life history strategies via the microbiota. However, because diapause status was not directly measured, this connection remains an inference. If validated, these microbial changes could provide a basis for developing novel pest management strategies by targeting the processes associated with diapause induction.

## Simple Summary

Photoperiod and temperature are key cues for insect diapause, and they interact to influence gut bacteria. In this study, *Loxostege sticticalis* larvae were exposed to different photoperiods (L10:D14 and L16:D8) and temperatures (18, 22, 26, 30 °C). Different combinations altered the microbial community non-linearly. The most dramatic shift occurred under a short photoperiod at 26 °C. Bacterial diversity dropped sharply, and *Enterococcus* dominated (99.96%). This change also predicted shifted bacterial activities toward carbohydrate breakdown and away from protein and energy processing. Other combinations also changed the gut bacteria but less extremely. These results suggest that photoperiod and temperature jointly change the gut bacterial community, which is hypothesized to help prepare the insect for winter dormancy (diapause). Understanding this process could lead to new ways to predict pest outbreaks or to develop environmentally friendly pest control strategies that target the pest’s gut microbes.

## 1. Introduction

Insect guts host a rich variety of microorganisms such as bacteria, fungi and archaea, which play vital roles in host nutrition, metabolism, immune defense, growth, development, and stress adaptation [1]. They enable insects to break down recalcitrant dietary components, produce indispensable amino acids and vitamins, detoxify harmful compounds, and resist pathogenic infection [2,3]. For example, the gut microbiota of *Drosophila melanogaster* influences nutritional homeostasis by regulating the insulin signaling pathway [4]. Microbial communities in *Bombyx mori* guts contribute to amino acid anabolism and enhance nutrient absorption efficiency [5]. Yeast symbionts associated with *Nilaparvata lugens* supply the host with required amino acids and B vitamins [6]. Numerous studies have shown that dynamic changes in gut microbiota are closely associated with insect health status, environmental adaptability, and population outbreaks [7,8,9]. Exploring gut microbial characteristics can clarify the ecological adaptation mechanisms of insects and also offer theoretical support for the development of innovative pest control tactics.

Diapause is a crucial adaptive strategy adopted by many insects to survive harsh environments including cold climates and food shortage [10]. Insect diapause is categorized into obligatory and facultative types, with environmental cues such as photoperiod and temperature primarily triggering the facultative diapause process [11,12]. Insects entering diapause usually need to build up large lipid reserves to sustain themselves [13]. Many studies have found that gut microorganisms help regulate insect diapause, especially in nutrient allocation and metabolism [14]. Under diapause, physiological adjustments in insect bacterial hosts often lead to the decline or even loss of specific gut bacterial taxa, thereby altering the overall structure of the gut microbial community. For example, in *Bombus impatiens*, gut microbial abundance drops by an order of magnitude during overwintering diapause, and the core genera *Schmidhempelia* and *Snodgrassella* become hard to detect later in diapause, though the overall metabolic potential remains stable [15]. Gut bacteria removal in diapause-destined *Helicoverpa armigera* reduces growth and increases feeding, confirming their role in diapause preparation via nutrient use [16]. These findings highlight that gut microorganisms act as a critical regulatory factor in the physiological preparation of insects for diapause.

*Loxostege sticticalis* is an important agricultural migratory pest widely distributed between 36° N and 54° N. It frequently breaks out in agricultural and pastoral areas of northern China by means of diapause and long-distance migration, causing enormous economic losses and ecological damage [17]. *L. sticticalis* overwinters as mature larvae inside cocoons, with diapause lasting up to eight months [18]. It is known that photoperiod plays a dominant role in diapause induction while temperature acts in conjunction. For example, short-day conditions (e.g., L10:D14) effectively induce diapause, whereas high temperatures significantly reduce diapause rates and shorten the critical day length [18,19]. Wang et al. found that prediapause *L. sticticalis* larvae differ from nondiapause larvae in gut bacterial community and have higher carbohydrate metabolism, supporting energy reserves during diapause [20]. However, that study focused only on photoperiod as a single factor and did not address temperature or its combined effect with photoperiod. In fact, temperature and photoperiod often jointly affect insect development and diapause progression [18,21], but how they synergistically regulate the structure and function of the gut microbiota in *L. sticticalis* remains unknown. Based on previous findings, we hypothesize that the gut microbiota is shaped by the combined effects of photoperiod and temperature, and may be involved in diapause preparation. To test this hypothesis, in this study we set up two photoperiods (L10:D14 and L16:D8) and four temperatures (18, 22, 26, and 30 °C), giving a total of eight treatment combinations (G10.18, G10.22, G10.26, G10.30, G16.18, G16.22, G16.26 and G16.30). Using 16S rRNA gene sequencing, we analyzed the composition, diversity, and functional potential of gut bacterial communities in 4th-instar larvae under these different light and temperature conditions. These results will help reveal how light and temperature affect the gut microecology of *L. sticticalis* and provide a theoretical basis for understanding its environmental adaptation and for developing pest control strategies based on gut microbiota regulation.

## 2. Materials and Methods

### 2.1. Insect Rearing

*L. sticticalis* larvae were originally collected from Zhenglan Banner, Xilin Gol League, Inner Mongolia Autonomous Region, in 2024 and subsequently reared for more than 10 generations at the Plant Protection and Quarantine Station of Kangbao County, Hebei Province, China. In 2025, we obtained them from that station. After the larvae emerged as adults, newly emerged adults were placed in insect rearing cages (length × width × height: 30 cm × 30 cm × 30 cm). A cotton ball soaked with 5% honey solution was placed at the bottom of each cage for adult feeding, and parchment paper was placed inside the cage for oviposition. Eggs were collected daily and transferred to new rearing cages. After larval hatching, individuals were fed with sugar beet leaves. A 5 cm-thick layer of sterile soil was spread at the bottom of the cage to allow mature larvae to form cocoons and pupate. The rearing conditions for both larvae and adults were temperature 22 ± 2 °C, relative humidity 70% ± 5%, photoperiod L16:D8 (16 h light:8 h dark), and light intensity 18,000 lx. Prior to the experimental treatments, these larvae had been maintained under the same photoperiod and temperature for two consecutive generations.

### 2.2. Temperature and Photoperiod Treatments and Sample Collection in L. sticticalis

The experiment included two photoperiods (L10:D14 and L16:D8) and four temperatures (18, 22, 26, 30 °C), resulting in a total of eight treatment combinations. Newly hatched larvae of *L. sticticalis* were placed under each treatment condition, and their developmental status was observed daily until they reached the fourth instar, at which point they were used for gut dissection. Prior to dissection, the larvae were starved for 2 h, then immersed in 75% alcohol for 10 s, followed by rinsing with distilled water. In a clean bench, each larva was placed on a sterile Petri dish on ice for dissection. The gut was carefully removed and transferred into a sterile 1.5 mL centrifuge tube. For each treatment combination, 15 larvae were collected per replicate, and their guts were pooled into a single sample; four such replicate samples were collected per treatment, giving a total of 60 larvae per treatment combination. Samples were labelled using a code combining photoperiod and temperature (e.g., “G10.26” for the L10:D14 at 26 °C treatment, “G16.18” for L16:D8 at 18 °C, and so on), with replicate numbers appended (e.g., G10.26.1 to G10.26.4). This yielded 32 samples in total (8 treatments × 4 replicates). After collection, the samples were immediately flash-frozen in liquid nitrogen and then transferred to a −80 °C freezer for storage until subsequent experiments.

### 2.3. Genomic DNA Extraction

Genomic DNA was extracted from the gut samples of *L. sticticalis* collected under different temperature and photoperiod treatments using the cetyltrimethylammonium bromide (CTAB) method. Subsequently, the purity and concentration of the DNA were assessed by 1% agarose gel electrophoresis. An appropriate amount of the sample was placed in a centrifuge tube and diluted with sterile water to a final concentration of 1 ng/μL. In each PCR run, no-template controls (NTCs) using ddH_2_O were included as negative controls. Only when no bands were detected in the NTCs were subsequent experiments carried forward.

### 2.4. PCR Amplification and High-Throughput Sequencing of Bacterial 16S rRNA Genes

Using the extracted genomic DNA as a template, the V3–V4 region of the bacterial 16S rRNA gene was amplified with the universal primers 341F (5′-CCTAYGGGRBGCASCAG-3′) and 806R (5′-GGACTACNNGGGTATCTAAT-3′). The PCR reaction system (30 μL) consisted of: Phusion Master Mix (2×) 15 μL, forward and reverse primers (2 μmol/L) 1.5 μL each, template gDNA (1 ng/μL) 10 μL, and ddH_2_O 2 μL. The amplification program was: initial denaturation at 98 °C for 1 min; followed by 30 cycles of denaturation at 98 °C for 10 s, annealing at 50 °C for 30 s, and extension at 72 °C for 30 s; and a final extension at 72 °C for 5 min. Samples were mixed in equal amounts based on PCR product concentration. After thorough mixing, the PCR products were subjected to electrophoresis on a 2% agarose gel, and bands of 400–450 bp were excised and purified using a GeneJET Gel Extraction Kit (Thermo Scientific, Waltham, MA, USA). After verification of concentration and specificity, the purified products were sent to Beijing Novogene Technology Co., Ltd. (Beijing, China) for high-throughput sequencing. Paired-end sequencing was performed on the Illumina MiSeq PE250 platform (Illumina, San Diego, CA, USA).

### 2.5. Data Processing and Analysis

Based on the 16S rRNA amplicon sequencing data, the composition, structure, and function of the gut microbial community of *L. sticticalis* under different temperature and photoperiod treatments were analyzed. The data preprocessing steps were as follows. First, the sample data were demultiplexed. Then, Cutadapt software (v3.3) was used to remove barcode and primer sequences from the reads. After trimming, paired-end reads were assembled using FLASH software (v1.2.11, http://ccb.jhu.edu/software/FLASH/ (accessed on 13 October 2025)) to obtain raw tag sequences [22]. Following assembly, stringent filtering was applied to obtain high-quality tag sequences. Chimeric sequences were detected by comparison with the Silva database (https://www.arb-silva.de/ (accessed on 13 October 2025)) and removed to obtain the final effective data. The DADA2 algorithm (v1.16) was used to infer amplicon sequence variants (ASVs) from the effective sequences [23]. To correct for differences in sequencing depth across samples, all samples were rarefied to 42,909 sequences per sample prior to the calculation of alpha and beta diversity indices. Rarefaction curves were generated to confirm that the sequencing depth was sufficient to capture the majority of ASV richness (ASVs are exact sequence variants and do not strictly equate to bacterial species) in all samples. Taxonomic annotation was performed based on the Silva database to obtain taxonomic information at the phylum, class, order, family, genus, and species levels [24]. ASV-based sample complexity (alpha-diversity, *α*-diversity) and multi-sample comparative analysis (Beta-diversity, *β*-diversity) were performed using Qiime software (v1.9.1). *β*-diversity was visualized using principal coordinate analysis (PCoA) based on Bray–Curtis distances, and dissimilarity tests based on adonis (PERMANOVA) were used to evaluate differences among treatment groups. For alpha diversity (*α*-diversity), the Shannon index, Simpson index, and Chao1 index were subjected to Levene’s test for homogeneity of variance and the Shapiro–Wilk test for normality. As the data did not meet the assumptions of normality or homogeneity of variance, the Scheirer-Ray-Hare test was used to evaluate the main effects of photoperiod and temperature as well as their interaction on *α*-diversity indices. The Kruskal–Wallis method was used to analyze differences in the above indices among treatment groups; when significant differences were detected, Dunn’s post-hoc test was applied with Benjamini–Hochberg correction for multiple comparisons. For beta diversity, a two-way PERMANOVA was performed to test the main effects of photoperiod, temperature, and their interaction on gut bacterial community structure based on Bray–Curtis distances with 999 permutations. *β*-diversity was visualized using principal coordinate analysis (PCoA) based on Bray–Curtis distances. Linear Discriminant Analysis (LDA) Effect Size (LEfSe) was used to identify differentially abundant taxa among treatment groups, with an LDA threshold of >4. The function of the gut microbiota of *L. sticticalis* was predicted using PICRUSt2 (v2.3.0) against the Kyoto Encyclopedia of Genes and Genomes (KEGG) Orthology database (default database bundled with the software), following the standard pipeline implemented by Novogene Co., Ltd. (Beijing, China) KEGG functional annotation was performed for each ASV. Species-level taxonomic annotation was performed using the Silva database (version 138.2). The quality of functional predictions was assessed using the weighted Nearest Sequenced Taxon Index (NSTI), with an average value of 0.019 across all samples (range: 0.00018–0.06846), indicating excellent prediction reliability according to PICRUSt2 quality guidelines. All data analyses and visualizations were performed using R (version 4.2.1).

## 3. Results

### 3.1. Species Annotation and Assessment of Gut Bacteria in L. sticticalis Larvae Under Different Photoperiod and Temperature Treatments

**Figure 1:**
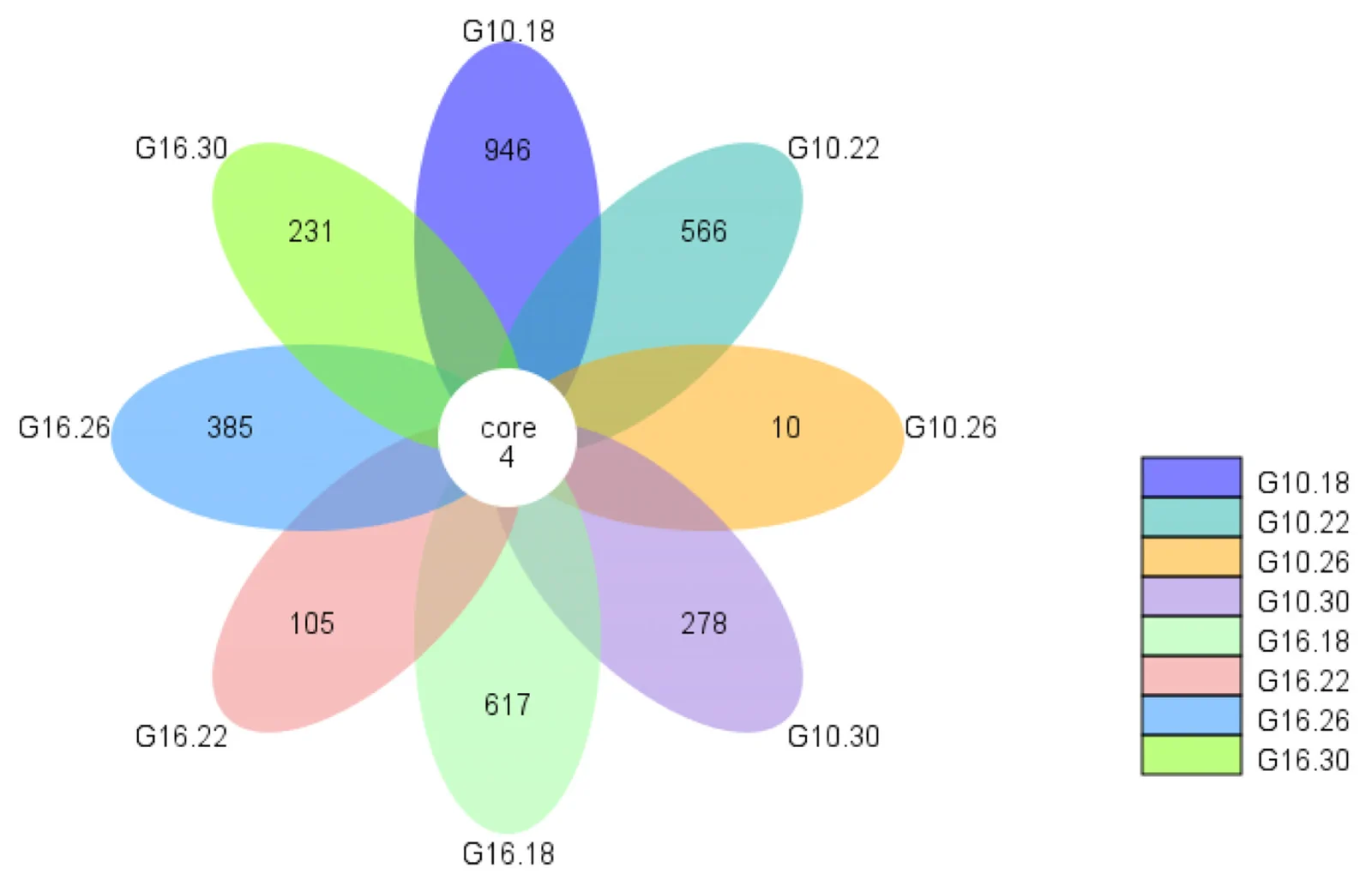
Unique and shared features composition of gut microbiota in *L. sticticalis* larvae under different photoperiod and temperature treatments. Each petal corresponds to a group, and distinct colors distinguish different groups. The central core number shows the count of feature sequences common to all groups, whereas the numbers on the petals indicate the count of feature sequences exclusive to each respective group. G10.18: photoperiod L10:D14 at 18 °C; G10.22: photoperiod L10:D14 at 22 °C; G10.26: photoperiod L10:D14 at 26 °C; G10.30: photoperiod L10:D14 at 30 °C; G16.18: photoperiod L16:D8 at 18 °C; G16.22: photoperiod L16:D8 at 22 °C; G16.26: photoperiod L16:D8 at 26 °C; G16.30: photoperiod L16:D8 at 30 °C. The same below.

**Figure 2:**
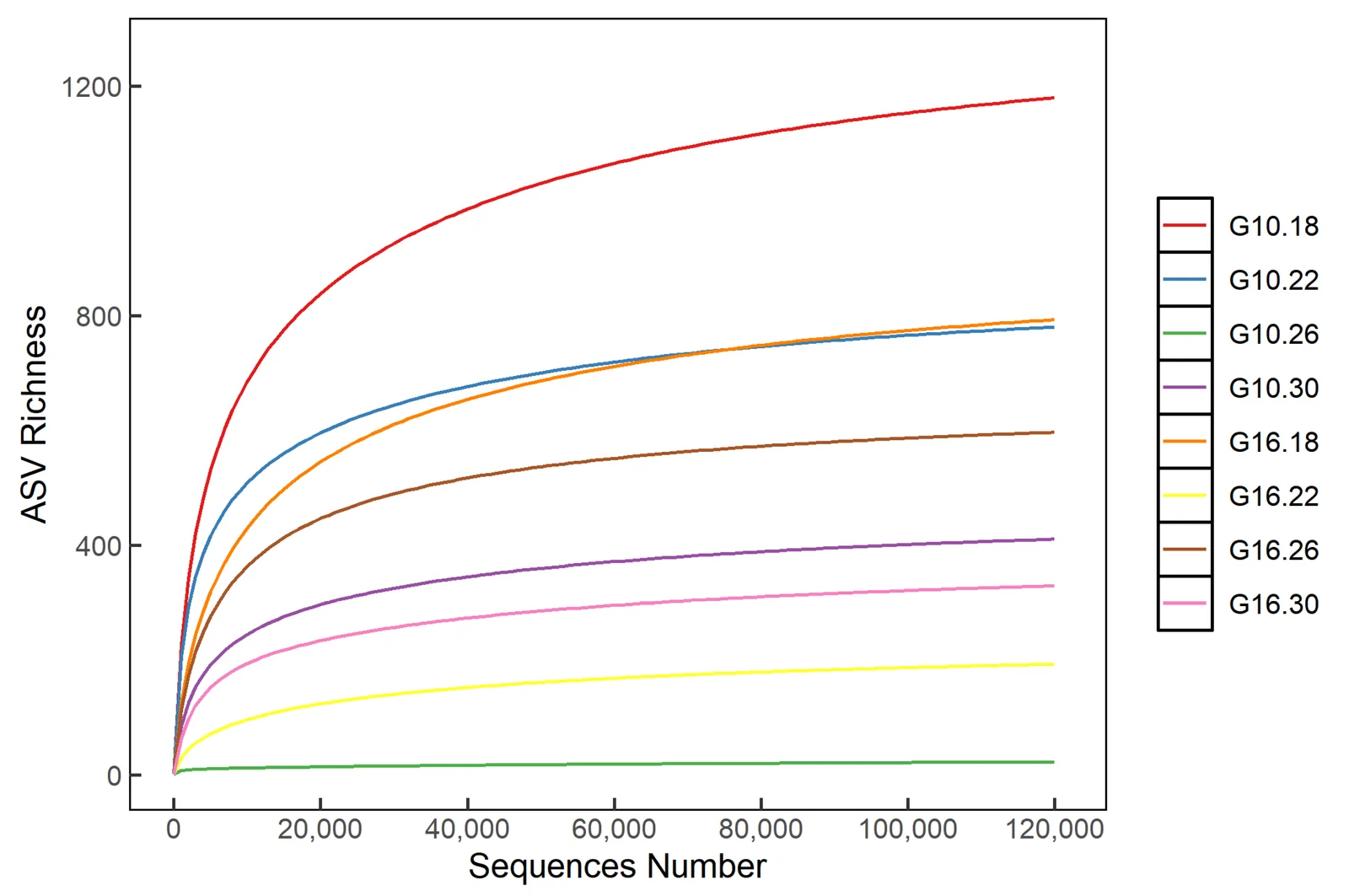
Rarefaction curves of gut bacterial composition in *L. sticticalis* under different photoperiod and temperature treatments.

A total of 3,283,343 raw 16S rRNA gene sequences were obtained from 32 intestinal samples of *L. sticticalis* larvae subjected to different photoperiod and temperature treatments. After quality filtering and chimera removal, 3,054,855 high-quality sequences were retained, with an average sequence length of 424 bp (Table S1). After rarefying to the minimum sequencing depth across samples, a total of 3645 ASVs were obtained, of which four appeared in all samples (Figure 1). Taxonomic annotation revealed that these ASVs belonged to 29 phyla, 52 classes, 128 orders, 205 families, 407 genera, and 281 ASVs at species-level taxonomic annotation (Table S2). Rarefaction curves showed that the number of observed ASVs in each treatment group tended to flatten and reach saturation as the number of sampled sequences increased, indicating that the sequencing data were reliable and could accurately reflect the ASV richness (Figure 2).

### 3.2. Diversity of Gut Bacterial Communities in L. sticticalis Larvae Under Different Photoperiod and Temperature Treatments

Alpha diversity indices of gut bacteria in *L. sticticalis* differed significantly among photoperiod and temperature treatments. Scheirer-Ray-Hare analysis further revealed significant interactive effects of photoperiod and temperature on Shannon, Simpson, and Chao1 indices (Table 1). Under L10:D14, significant differences were detected between 18 °C and 26 °C for Shannon (*H* = 9.860, df = 3, *p* = 0.020) and Chao1 (*H* = 10.096, df = 3, *p* = 0.018) indices, and between 22 °C and 26 °C for Simpson index (*H* = 10.213, df = 3, *p* = 0.017); no other pairwise differences were found among temperatures (Table 2). Under L16:D8, Shannon index differed significantly between 22 °C and 26 °C (*H* = 10.213, df = 3, *p* = 0.017), while no significant differences were observed for Simpson or Chao1 indices across temperatures (Table 2). Taken together, G10.26 treatment group had the lowest diversity, while G10.18 and G10.22 had the highest diversity, indicating that photoperiod and temperature have an interactive effect on the alpha diversity of gut microbiota in *L. sticticalis* larvae.

**Figure 3:**
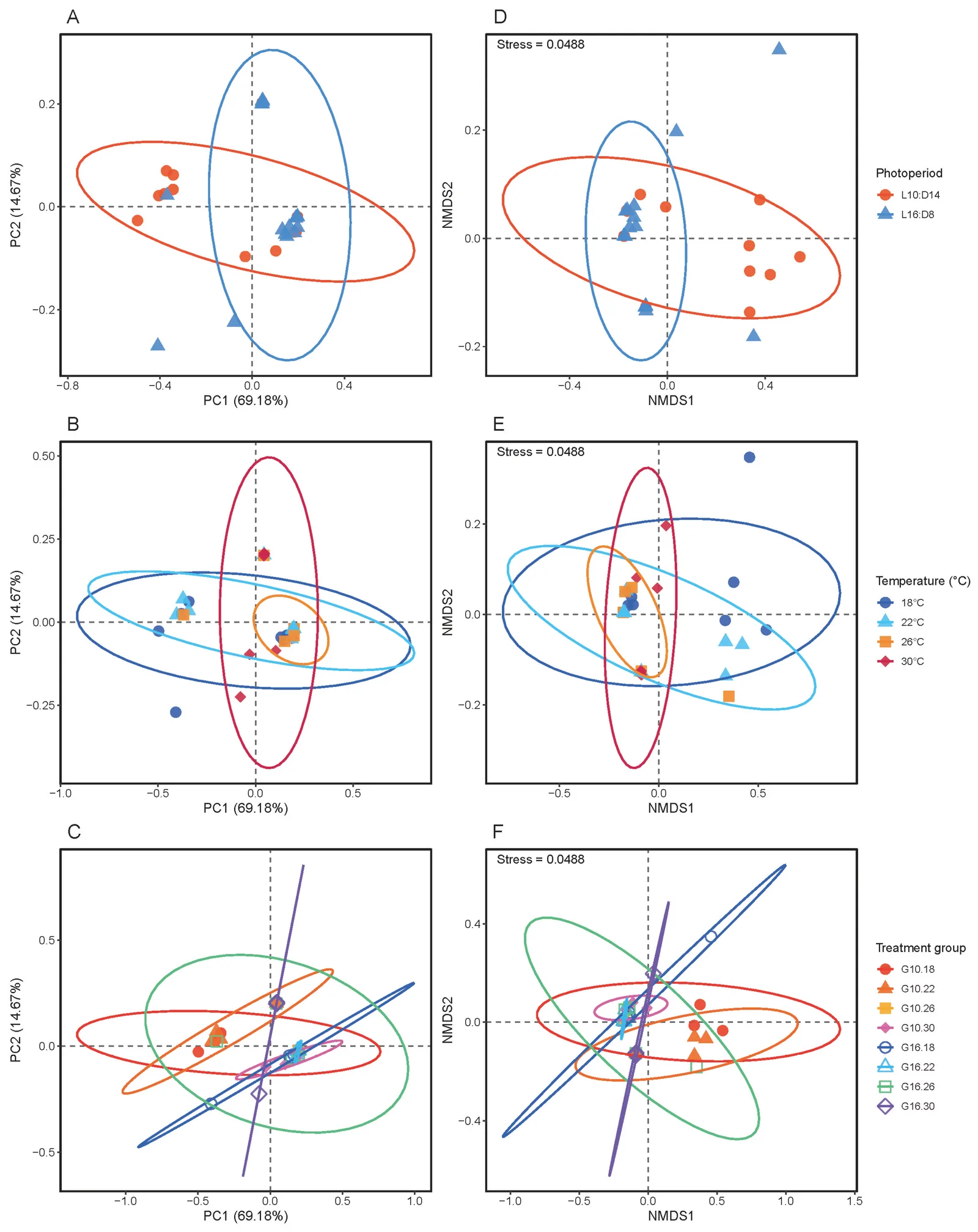
PCoA and NMDS analysis of gut microbiota in *L. sticticalis* under different photoperiod and temperature treatments based on Bray–Curtis distances. (**A**) PCoA of samples colored by photoperiod (L10:D14 vs. L16:D8). (**B**) NMDS of samples colored by photoperiod. (**C**) PCoA of samples colored by temperature (18, 22, 26, 30 °C). (**D**) NMDS of samples colored by temperature. (**E**) PCoA of samples colored by the eight treatment combinations (G10.18, G10.22, G10.26, G10.30, G16.18, G16.22, G16.26, G16.30). (**F**) NMDS of samples colored by the eight treatment combinations. Each point represents an individual sample, with different colors and shapes distinguishing treatment groups. Different color ellipses indicate 95% confidence intervals for each group. Stress values for NMDS ordinations are shown in the upper-left corner of each NMDS panel.

Beta diversity analysis was used to compare the gut microbiota composition of *L. sticticalis* under different photoperiod and temperature treatments based on Bray–Curtis distances. Principal coordinate analysis (PCoA) and non-metric multidimensional scaling (NMDS) ordinations revealed distinct clustering patterns among treatment groups (Figure 3). Two-way PERMANOVA revealed a significant interactive effect of photoperiod and temperature on the gut bacterial community structure (Table 3), confirming that the effect of temperature on community composition depends on photoperiod. In contrast, when examined separately, neither photoperiod nor temperature showed a significant effect on community composition (Table 3). Principal coordinate analysis (PCoA) based on Bray–Curtis distance showed that the contribution rates of principal coordinate 1 (PC1) and principal coordinate 2 (PC2) were 69.18% and 14.67%, respectively. Temperature was the dominant factor associated with community differentiation, and its effect was modulated by photoperiod. Under the L10:D14 photoperiod, the 26 °C treatment group (G10.26) was far from other temperature treatment groups under the same photoperiod (G10.18-G10.26: Adonis *R*^2^ = 0.526, *p* = 0.037; G10.22-G10.26: Adonis *R*^2^ = 0.630, *p* = 0.029; G10.26-G10.30: Adonis *R*^2^ = 0.734, *p* = 0.029) (Figure 3A and Table S3). Under the L16:D8 photoperiod, significant differences were only found between the two groups with the largest temperature spans, i.e., G16.18 vs. G16.30 (*R*^2^ = 0.488, *p* = 0.035) and G16.22 vs. G16.30 (*R*^2^ = 0.436, *p* = 0.038) (Figure 3 and Table S3). Regarding photoperiod effects, significant differences between L10:D14 and L16:D8 groups were detected at 26 °C (G10.26-G16.26: *R*^2^ = 0.545, *p* = 0.022) and 30 °C (G10.30-G16.30: *R*^2^ = 0.434, *p* = 0.048), but not at 18 °C or 22 °C (Figure 3 and Table S3). Similar to the PCoA results, non-metric multidimensional scaling (NMDS) analysis showed differences in the gut bacterial community structure of *L. sticticalis* under different photoperiod and temperature treatments [stress = 0.0488 (<0.2)] (Figure 3F). These results suggest that different photoperiods and temperatures greatly affect the gut bacterial community composition of *L. sticticalis* larvae.

### 3.3. Composition of Gut Bacterial Communities in L. sticticalis Larvae Under Different Photoperiod and Temperature Treatments

**Figure 4:**
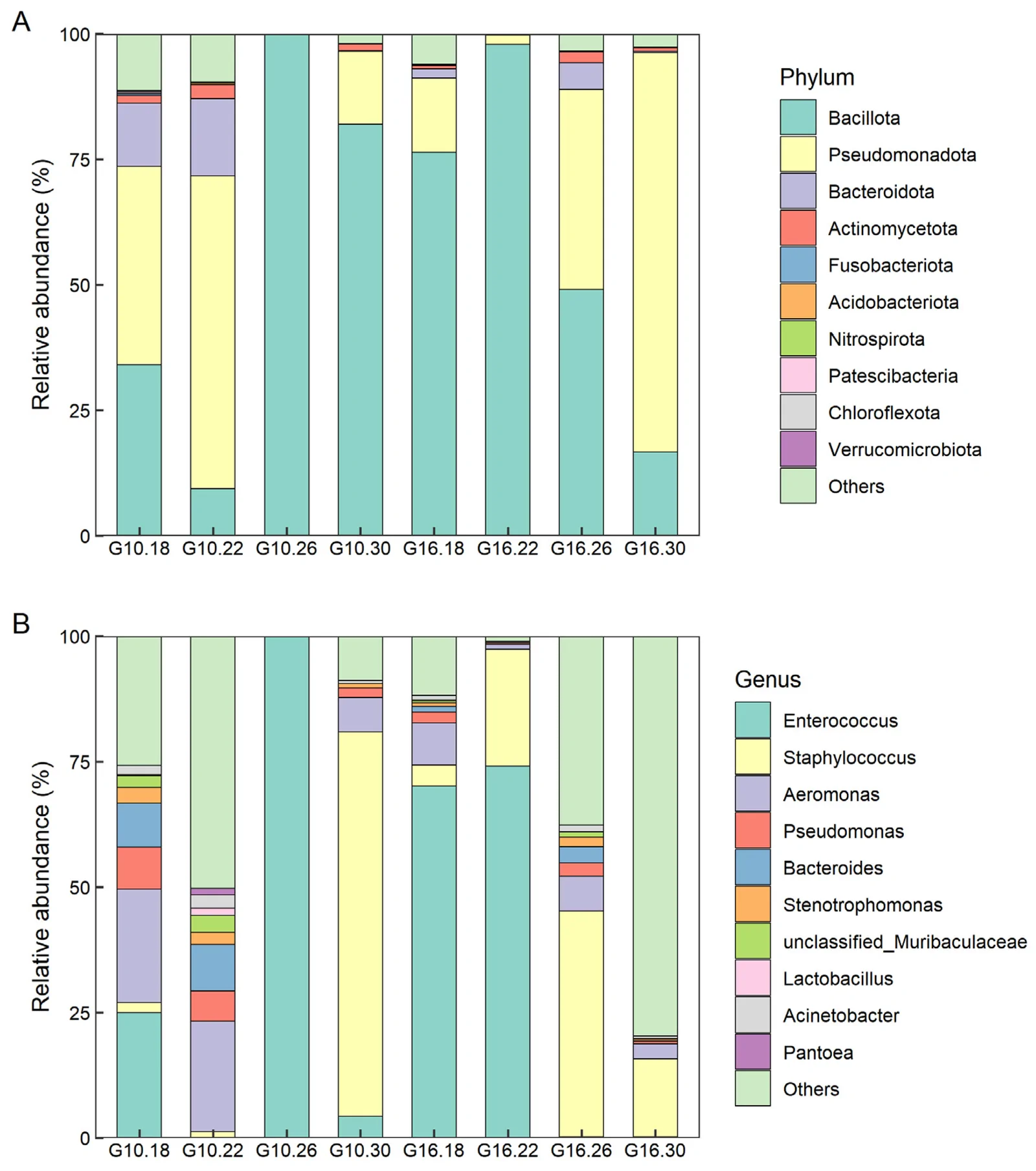
Gut bacterial composition at the phylum (**A**) and genus (**B**) levels in *L. sticticalis* larvae under different photoperiod and temperature treatments. Each color represents a phylum or genus, and “Others” indicates the sum of relative abundances of all other phyla (or genera) not shown among the 10 categories in the figure.

At the phylum level, under the photoperiod L10:D14, the dominant phylum in the midgut of larvae in the 18 °C (G10.18) and 22 °C (G10.22) treatment groups was Pseudomonadota, accounting for 39.54% and 62.30%, respectively (Figure 4A). The subdominant phylum in the G10.18 group was Bacillota (34.04%), while that in the G10.22 group was Bacteroidota (15.34%) (Figure 4A). In the 26 °C (G10.26) and 30 °C (G10.30) treatment groups, the dominant phylum in the larval gut was Bacillota, accounting for 99.98% and 81.99%, respectively. Notably, the G10.26 group lacked the following phyla: Bacteroidota, Actinomycetota, Fusobacteriota, Acidobacteriota, Nitrospirota, Patescibacteria, Chloroflexota, and Verrucomicrobiota (Figure 4A). Under the photoperiod L16:D8, the dominant phylum in the 30 °C (G16.30) group was Pseudomonadota (79.56%), and the subdominant phylum was Bacillota (16.66%) (Figure 4A). In the remaining temperature treatment groups (G16.18, G16.22, G16.26), the dominant phylum was Bacillota, accounting for 76.42%, 97.89%, and 49.04%, respectively. The subdominant phylum in these groups was Pseudomonadota, accounting for 14.70%, 1.79%, and 39.84%, respectively (Figure 4A).

At the genus level, the dominant genus in the G10.18, G10.26, G16.18 and G16.22 treatment groups was *Enterococcus*, with relative abundances of 25.02%, 99.96%, 70.12% and 74.16%, respectively (Figure 4B). This pattern was consistently observed across all four biological replicates in each of these treatment groups (Table S4). However, the subdominant genera differed among these groups. In the G10.18, G10.26 and G16.18 groups, the subdominant genus was *Aeromonas*, accounting for 22.57%, 8.39% and 0.01%, respectively (Figure 4B). In the G16.22 group, the subdominant genus was *Staphylococcus*, accounting for 23.22% (Figure 4B). The dominant genus in the G10.30, G16.26 and G16.30 groups was *Staphylococcus*, with relative abundances of 76.65%, 44.98% and 15.70%, respectively (Figure 4B). The subdominant genus in these groups was *Aeromonas*, accounting for 6.82%, 7.04% and 3.00%, respectively (Figure 4B). In the G10.22 group, the dominant genus was *Aeromonas* (22.08%), followed by *Bacteroides* (9.23%) (Figure 4B). Notably, the G10.18, G10.26, G10.30, G16.22 and G16.30 groups all lacked the genus *Pantoea*. In addition, the G10.26 group lacked several genera, including *Staphylococcus*, *Pseudomonas*, *Bacteroides*, *Stenotrophomonas*, and *Lactobacillus*. The G16.22 group lacked the genus *Bacteroides*. The G10.30 group lacked two genera, unclassified *Muribaculaceae* and *Lactobacillus*.

**Figure 5:**
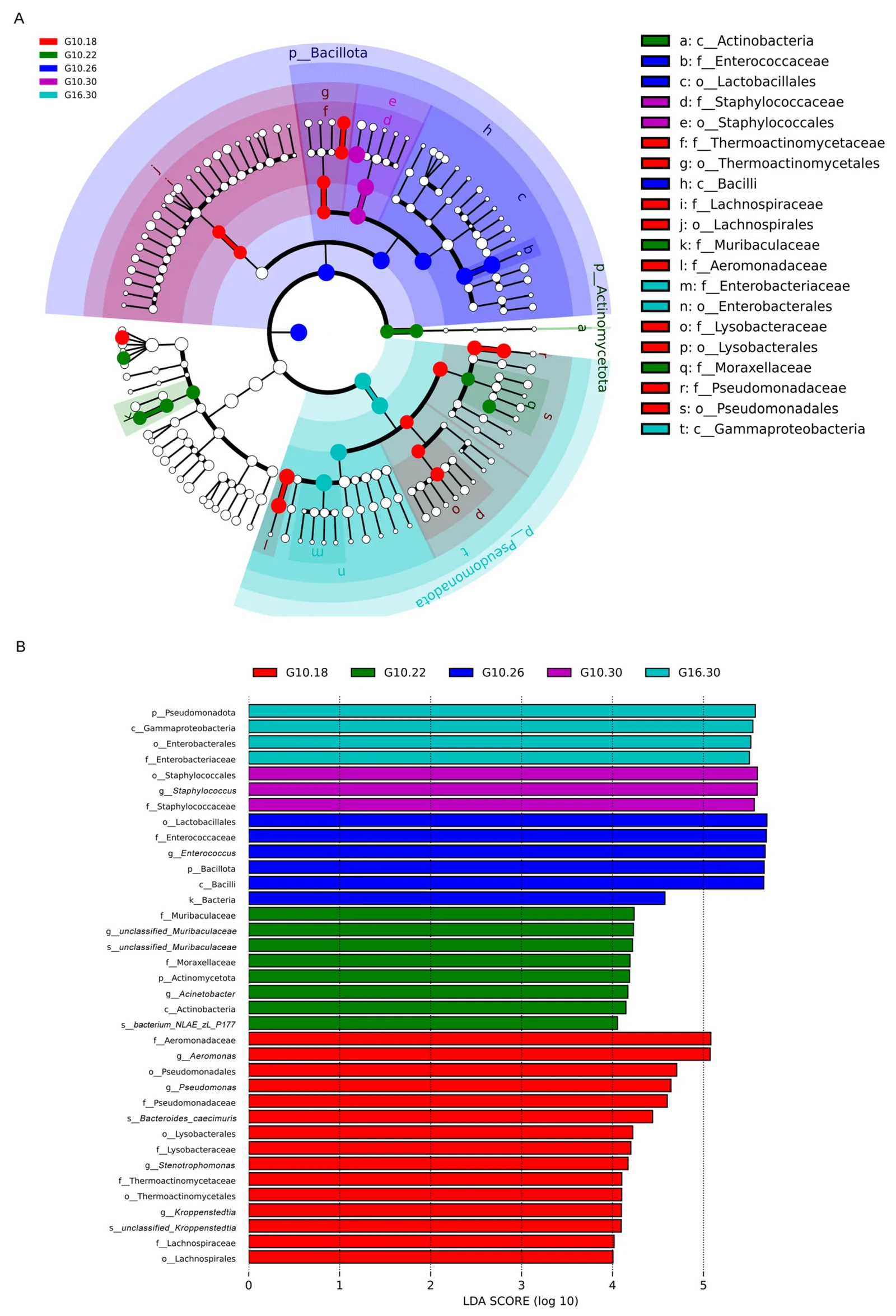
LEfSe analysis of gut bacterial communities in *L. sticticalis* larvae under different photoperiod and temperature treatments. (**A**) represents the LEfSe analysis cladogram. Cladogram showing the taxonomic hierarchy from phylum to genus. Circles radiating from the inside to the outside represent taxonomic levels; each circle at a given level represents a taxon, and its diameter is proportional to the relative abundance. Non-significant taxa are colored white, while biomarkers with significant differences are colored according to the treatment group in which they are enriched. (**B**) Bar chart of LDA scores (LDA > 4). Note that groups without biomarkers (G16.18, G16.22, G16.26) are absent from the plot.

LEfSe analysis was used to identify characteristic microbial taxa associated with photoperiod and temperature in the gut bacterial communities of *L. sticticalis* larvae under different treatments. Under the screening threshold set in this study (LDA > 4), 20 biomarkers with significant differences among groups were identified. Only five groups (G10.18, G10.22, G10.26, G10.30, and G16.30) showed representative biomarkers meeting the significant enrichment threshold, while the other three groups (G16.18, G16.22, and G16.26) did not detect any characteristic taxonomic units meeting the screening threshold (Figure 5). At the phylum level, Actinomycetota was significantly enriched in the G10.22 group. Bacillota was significantly enriched in the G10.26 group. Pseudomonadota was significantly enriched in the G16.30 group. At the class level, Actinobacteria was significantly enriched in the G10.22 group, Bacilli was significantly enriched in the G10.26 group, Gammaproteobacteria was significantly enriched in the G16.30 group. At the order level, Pseudomonadales, Lysobacterales, Thermoactinomycetales, and Lachnospirales were significantly enriched in the G10.18 group, Staphylococcales was significantly enriched in the G10.30 group, Enterobacterales was significantly enriched in the G16.30 group. At the family level, Aeromonadaceae, Pseudomonadaceae, Lysobacteraceae, Thermoactinomycetaceae, and Lachnospiraceae were significantly enriched in the G10.18 group, Muribaculaceae and Moraxellaceae were significantly enriched in the G10.22 group, Enterococcaceae was significantly enriched in the G10.26 group, Staphylococcaceae was significantly enriched in the G10.30 group, Enterobacteriaceae was significantly enriched in the G16.30 group. At the genus level, Aeromonas, Pseudomonas, Stenotrophomonas, and Kroppenstedtia were significantly enriched in the G10.18 group. Acinetobacter was significantly enriched in the G10.22 group, *Enterococcus* was significantly enriched in the G10.26 group, Staphylococcus was significantly enriched in the G10.30 group. Sensitivity analysis using the default LDA > 2 confirmed that the key biomarkers, including *Enterococcus* (Figure S1), remained significant, indicating the robustness of our findings to threshold selection.

### 3.4. Functional Prediction of Gut Bacteria in L. sticticalis Larvae Under Different Photoperiod and Temperature Treatments

**Figure 6:**
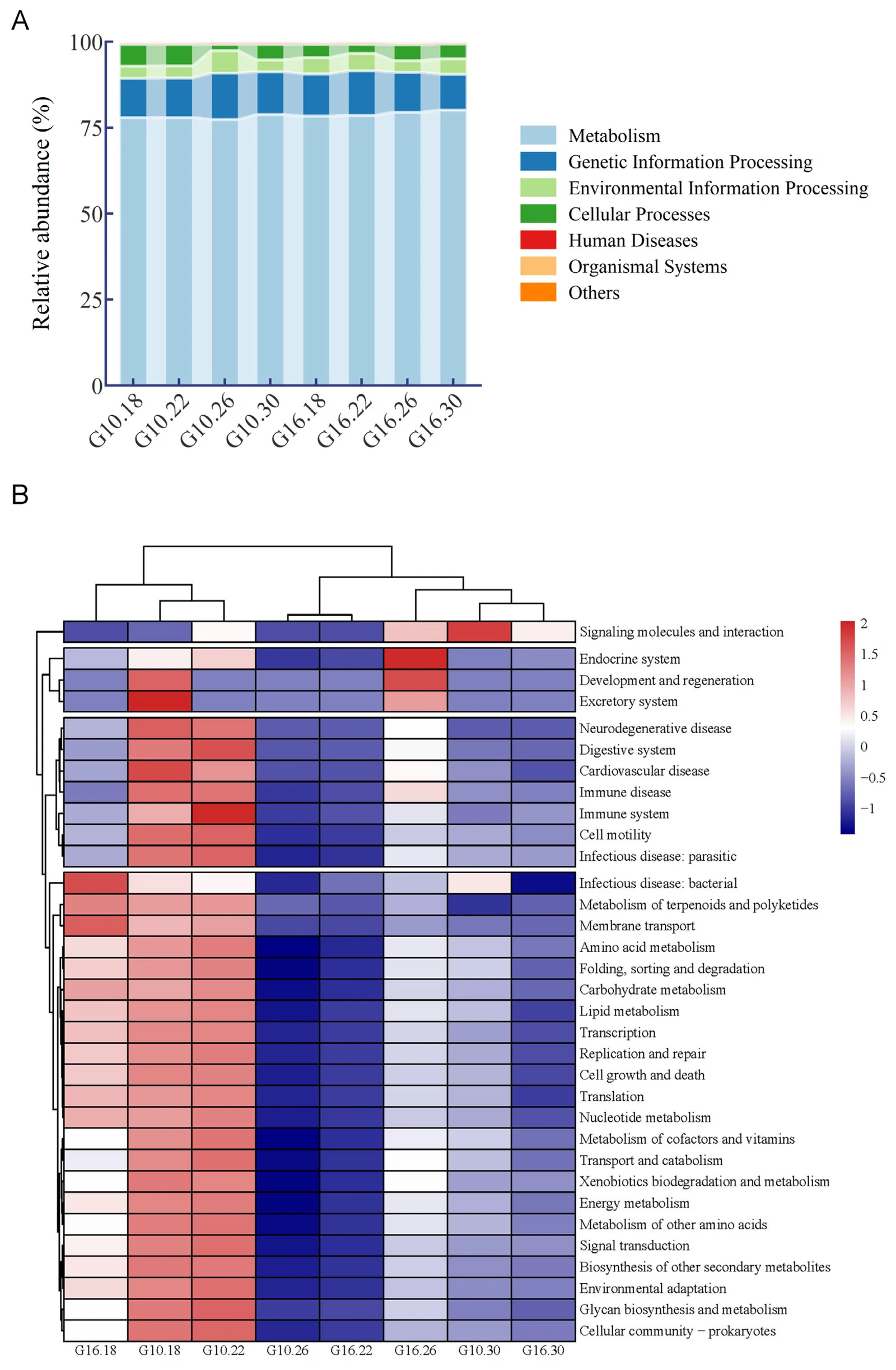
Predicted functional profiles of gut bacterial communities in *L. sticticalis* larvae under different photoperiod and temperature treatments, inferred using PICRUSt2 (v2.3.0) against the KEGG database. (**A**) Relative abundance of KEGG pathways at level 1. (**B**) Heatmap showing the relative abundance of the top 10 KEGG pathways at level 2. The clustering tree on the left side of the figure is a species clustering tree, and the clustering tree at the top represents the clustering of treatment groups. The values displayed in the heatmap correspond to the Z-scores obtained after row-wise standardization of the relative abundance of each species (or taxon). Specifically, the Z-score for a given sample at a given taxon is calculated as the difference between the relative abundance of that taxon in that sample and the mean relative abundance of that taxon across all samples, divided by the standard deviation of that taxon across all samples.

Functional prediction based on 16S sequencing data using PICRUSt2 and the KEGG database suggested that the gut microbiota of *L. sticticalis* larvae under different photoperiod and temperature treatments could be annotated into six functional categories at KEGG level 1 (Figure 6A). The core function of all treatment groups was primarily metabolism (relative abundance > 76% in all groups), but variations were observed among groups in the relative abundance of specific functional categories. The relative abundance of the metabolism pathway in the G10.26 group was significantly lower than that in the G16.30 group (*H* = 16.920, df = 7, *p* = 0.018). The relative abundance of the cellular processes pathway in the G10.26 group was significantly lower than that in the G10.22 group (*H* = 15.102, df = 7, *p* = 0.035). However, the relative abundance of the genetic information processing pathway in the G10.26 group was significantly higher than that in the G16.30 group (*H* = 21.000, df = 7, *p* = 0.004), and the relative abundance of the environmental information processing pathway in the G10.26 group was significantly higher than that in the G16.26 group (*H* = 17.989, df = 7, *p* = 0.012).

Functional annotation of the gut microbiota of *L. sticticalis* larvae under different photoperiod and temperature treatments indicated 33 functional categories at KEGG level 2. The top 10 pathways in relative abundance were carbohydrate metabolism, metabolism of cofactors and vitamins, amino acid metabolism, metabolism of terpenoids and polyketides, xenobiotics biodegradation and metabolism, lipid metabolism, energy metabolism, metabolism of other amino acids, replication and repair, and glycan biosynthesis and metabolism (Figure 6B). For carbohydrate metabolism, the relative abundance of gut bacteria in the G10.26 group was significantly higher than that in the G10.22 group. The G16.22 group also had significantly higher abundance than the G10.18 group (*H* = 17.847, df = 7, *p* = 0.013). In the G10.26 group, the abundances of metabolism of cofactors and vitamins, amino acid metabolism, replication and repair, and energy metabolism were significantly lower than those in the G10.30 group. The Energy metabolism abundance in G10.26 was also significantly lower than that in the G16.26 group (*H* = 15.955, df = 7, *p* = 0.026). In contrast, the G10.26 group showed significantly higher abundance in metabolism of terpenoids and polyketides than the G10.30 group (*H* = 18.790, df = 7, *p* = 0.009). The G10.26 group also had significantly higher abundances in xenobiotics biodegradation and metabolism and metabolism of other amino acids than the G10.22 group (*H* = 20.369, df = 7, *p* = 0.005; *H* = 20.608, df = 7, *p* = 0.004). These results indicate that different photoperiod and temperature treatments may influence multiple metabolic functions of the gut microbiota in *L. sticticalis* larvae as predicted by KEGG annotation. The G10.26 group exhibited relatively higher carbohydrate metabolism potential but lower levels in cofactor metabolism, amino acid metabolism, and energy metabolism. In contrast, the G10.30 and G16.26 groups exhibited opposite trends. This suggests that the interaction between photoperiod and temperature plays an important regulatory role in shaping the predicted functional profile of the gut microbiota.

## 4. Discussion

This study found that both photoperiod and temperature were associated with significant changes in the composition and function of the gut bacterial community in *L. sticticalis* larvae, with an interactive effect between the two factors. Under the short-day photoperiod (L10:D14) condition, the 26 °C treatment group (G10.26) exhibited a marked decrease in gut bacterial α-diversity, and the relative abundance of *Enterococcus* reached 99.96%. Functional prediction indicated that the carbohydrate metabolism pathway showed higher predicted abundance in this treatment group, while the pathways of amino acid metabolism, metabolism of cofactors and vitamins, and energy metabolism showed lower predicted abundance. In contrast, under the long-day photoperiod (L16:D8), no significant differences in bacterial diversity were observed among the different temperature treatment groups, and no similar simplification occurred. These results indicate that specific combinations of photoperiod and temperature are associated with the structure and function of the gut bacterial community in *L. sticticalis* larvae.

Alpha diversity analysis showed that under short-day photoperiod, temperature had a non-linear effect on bacterial richness and evenness. The G10.18 and G10.22 treatment groups maintained relatively high Shannon and Chao1 indices, while both indices significantly decreased in the G10.26 group. Under a long-day photoperiod, no significant differences were observed among temperature treatment groups. This pattern was generally consistent with the diapause rate trend reported by Huang et al. [18], although diapause status was not directly measured in the present study. The G10.26 treatment group exhibited the most drastic reduction in diversity, suggesting that this specific photoperiod–temperature combination may impose strong selective pressure on the bacterial community, leading to extreme enrichment of *Enterococcus*. Notably, within the range of 18–30 °C, the developmental duration of *L. sticticalis* shortened with increasing temperature [25,26,27]. At 26 °C, larvae developed faster and exhibited vigorous feeding activity [27]. The G10.26 treatment group fell exactly into this sensitive and metabolically active stage, which may explain the drastic restructuring of the bacterial community. Wang et al. [20] also observed a significant decrease in α-diversity of diapause-destined larvae at 21 °C, consistent with our results. However, the most drastic simplification occurred at 26 °C in our study, indicating that temperature plays a key modulatory role in microbiota restructuring. Beta diversity analysis showed that the bacterial community structure of the G10.26 treatment group was significantly separated from all other treatment groups, while the structures of different temperature groups under long-day photoperiod were relatively clustered. This pattern suggests that the specific combination of short-day photoperiod and 26 °C induced a unique bacterial community structure. The significant separation in beta diversity between presumed diapause-destined and non-diapause larvae further supports the potential role of photoperiod in shaping bacterial community structure [20].

Photoperiod is an important environmental factor affecting the gut microbiota of insects. In *Bactrocera dorsali*, continuous light or continuous dark treatment significantly reduced gut bacterial diversity and altered the bacterial composition [28]. Temperature also affects gut microbiota, as seen in *Drosophila melanogaster*, where long-term exposure to different temperatures led to systematic changes in bacterial composition [29]. In *L. sticticalis*, photoperiod is the dominant factor inducing diapause, with temperature acting in concert, and their interaction is significant [18]. In the present study, under the short-day photoperiod, the gut microbiota was simplified at 26 °C but dominated by Staphylococcus at 30 °C; under the long-day photoperiod, no simplification occurred, but the dominant genera shifted with temperature. This indicates that gut microbiota simplification is not driven solely by photoperiod or temperature alone, but may be triggered by the diapause preparation state induced synergistically by both factors. LEfSe analysis identified characteristic biomarkers, distributed across five treatment groups: G10.18, G10.22, G10.26, G10.30, and G16.30. The biomarker for G10.26 was *Enterococcus*, a Gram-positive, facultatively anaerobic bacterium that is highly tolerant to environmental stress and possesses efficient carbohydrate fermentation capacity. Zhang et al. [30] confirmed that *Enterococcus* is the absolute dominant bacterium in the gut of *Phthorimaea operculella*, and reducing its abundance leads to decreased larval carbohydrate metabolism, lower survival rate, prolonged developmental duration, and reduced fecundity. *Enterococcus* exhibited the ability to promote faster growth in *Spodoptera frugiperda* larvae [31]. Therefore, the high abundance of *Enterococcus* in G10.26 raises the hypothesis that this bacterium may actively participate in the host’s metabolic shift. Moreover, in *Bombus impatiens* queens, Gram-negative bacteria were not detected during late diapause while Gram-positive bacteria persisted [15], which is consistent with the absolute dominance of Gram-positive *Enterococcus* in our study. This difference may be attributed to variations between Gram-negative and Gram-positive bacteria in cold sensitivity, lower thermal limits for growth, and susceptibility to changes in available exogenous nutrients and host metabolites [32,33,34], suggesting that Gram-positive bacteria may have a greater survival advantage under conditions associated with diapause-related environmental stress. The G10.18 group was enriched in *Aeromonas*, *Pseudomonas*, *Stenotrophomonas*, and *Kroppenstedtia*. *Aeromonas*, which was also subdominant in multiple groups, may represent a core, broad-tolerance gut bacterium; its functional diversity (environmental ubiquity, insecticide resistance, immune activation, antifungal enzyme production) is consistent with its persistence across treatments [35,36,37]. *Pseudomonas* produces antimicrobial substances [38], some *Stenotrophomonas* strains can degrade cellulose [39], and *Kroppenstedtia* produces hydrolytic enzymes such as amylase and protease [40]. The enrichment of these genera may thus be linked to nutritional metabolism regulation during conditions inferred to involve diapause preparation. The G10.22 group was enriched in *Acinetobacter* and the family Muribaculaceae. Some *Acinetobacter* strains can degrade lignocellulose and produce vitamin B12 [41]; Muribaculaceae is associated with short-chain fatty acid production and carbohydrate metabolism [42], and its enrichment may indicate a functional shift toward carbohydrate utilization. Under the short-day photoperiod (L10:D14) at 30 °C, the diapause rate of *L. sticticalis* was reported to be very low [18]. In our data under this same photoperiod-temperature combination, the gut microbiota was dominated by *Staphylococcus*, suggesting that the enrichment of this genus might mainly reflect the direct effect of high temperature on the gut microbiota rather than diapause adaptation. Similar temperature-induced shifts in gut bacterial communities, including an increase in *Staphylococcus*, have been observed in other insects (e.g., *Bombyx mori*) under heat stress [43]. The G16.30 group was enriched in the phylum Pseudomonadota, class Gammaproteobacteria, order Enterobacterales, and family Enterobacteriaceae, all of which are common commensals in insect guts and are associated with carbohydrate and energy metabolism [44]. Under long-day non-diapause conditions (L16:D8), larvae continuously feed and develop, and the gut microbiota maintains a diverse structure dominated by *Pseudomonadota*, in contrast to the simplified state in G10.26 under the inferred diapause preparation conditions.

During long-term evolution, insects and their symbiotic bacteria have developed complex mutualistic relationships: symbiotic bacteria not only participate in host nutritional metabolism but also extensively intervene in various life activities such as immune regulation and developmental regulation [7,45,46]. Based on functional prediction using 16S rRNA sequencing data using PICRUSt2, this study found that in the G10.26 treatment group, the relative abundance of the metabolism pathway at KEGG level 1 was significantly lower than that in the G16.30 group, the cellular processes pathway was lower than that in the G10.22 group, while the genetic information processing and environmental information processing pathways were higher than those in the G16.30 and G16.26 groups, respectively. At KEGG level 2, the G10.26 group exhibited significantly increased carbohydrate metabolism, while the metabolism of cofactors and vitamins, amino acid metabolism, and energy metabolism were significantly reduced. The increases and decreases in these pathways showed a covarying pattern, similar to the functional interdependence among different bacterial families in the *Drosophila* gut microbiota [29]. In the G10.26 group, the predicted metabolic shift toward enhanced carbohydrate metabolism and reduced amino acid and energy metabolism was the most pronounced among all treatment groups. At 26 °C, larvae are in an active stage of feeding and development [27]; the G10.26 group was exposed to a 10 h short-day photoperiod, which meets the conditions for diapause induction. Under such conditions, larvae are highly sensitive, and even if only a portion of individuals eventually enter diapause, all individuals experience strong physiological stress, which may impose stringent selective pressure on the gut microbiota, leading to extreme enrichment of *Enterococcus* and driving the metabolic shift toward carbohydrate utilization. In contrast, the G10.30 group, despite having a higher temperature, experienced a weaker diapause induction signal; its gut microbiota was dominated by Staphylococcus without metabolic simplification. The G10.18 group developed slowly with weak feeding activity and retained relatively high bacterial diversity. These observations indicate that the inferred metabolic reprogramming may require the interactive effect of 26 °C and a short-day photoperiod, i.e., maintaining vigorous feeding while perceiving diapause signals. This reprogramming may be a pre-adaptation process in which the host actively modulates the gut environment (e.g., altering pH, oxygen concentration, secreting antimicrobial peptides) to select for *Enterococcus*, rather than a direct effect of temperature or photoperiod. Wang et al. [20] also observed a similar metabolic shift under constant 21 °C with LD12:12 inducing diapause. In *Bombyx mori* and *Clanis bilineata tsingtauica*, *Enterococcus* abundance was negatively correlated with amino acid metabolism [47,48]. Based on these findings, it is hypothesized that *Enterococcus* may help larvae convert carbon sources into storage substances and reduce nitrogenous waste by downregulating amino acid synthesis and enhancing carbohydrate fermentation. This strategy of relying on gut microbiota to perform specific metabolic functions is also observed in diapausing *Parastrachia japonensis*. In contrast, bumblebee queens maintain stable metabolic potential during diapause [49]. Compared with these cases, the extreme simplification of the gut microbiota in *L. sticticalis* occurs only under the interactive conditions of a short-day photoperiod and 26 °C when larvae maintain vigorous feeding, and this may represent a special stress response triggered by the combination of high-temperature active metabolism and diapause induction signals. However, pooling 15 guts per replicate provided sufficient DNA for sequencing, a common practice in insect microbiome studies. This design averages individual profiles and may obscure inter-individual heterogeneity. Our four replicates per treatment were included to evaluate overall treatment-level average responses, but the pooled approach does not permit assessment of individual-level variation. Future studies with individual gut sequencing are needed to address this limitation. Beyond this methodological consideration, several additional limitations should be acknowledged. First, the present study sampled only at a single time point and thus cannot capture the temporal dynamics of microbiota changes during diapause preparation. Second, we examined the gut microbiota of 4th-instar larvae only, without distinguishing different developmental stages or monitoring the transition to subsequent instars. Third, and most importantly, we did not directly measure diapause physiological indicators (e.g., hormone levels, respiratory metabolic rate, fat body accumulation) in the experimental larvae; the diapause status of larvae was inferred solely from photoperiod-temperature combinations based on the previous report of Huang et al. [18]. In 3rd-instar presumed diapause-destined larvae of *L. sticticalis*, carbohydrate metabolism increased and amino acid metabolism decreased, whereas different trends were observed in 4th and 5th instars [20]. Therefore, the metabolic differentiation in the G10.26 group in the present study may reflect the effect of a specific photoperiod-temperature combination at a particular developmental stage, indicating complex interactions between microbiota changes and developmental stage. Future studies should adopt multi-time-point sampling combined with gut microbiota transplantation or targeted removal of key bacterial genera for causal validation.

## 5. Conclusions

In conclusion, this study shows that photoperiod and temperature are associated with the structure and function of the gut microbiota in *L. sticticalis* larvae. Notably, the combination of a short-day photoperiod (L10:D14) and 26 °C induced a distinct state dominated by *Enterococcus* with low diversity and a predicted metabolic shift favoring carbohydrate utilization. These findings raise the hypothesis that gut microbiota may participate in the metabolic reprogramming associated with insect diapause preparation and suggest how environmental factors (photoperiod and temperature) may mediate host life history strategies through the microbiota. However, because diapause status was not directly measured in this study, the connection between microbiota changes and diapause preparation remains an inference rather than a demonstrated fact. Moreover, monitoring specific changes in the gut microbiota could potentially serve as an early warning indicator for diapause onset in *L. sticticalis*, and targeted regulation of key microbial taxa may represent a novel ecological pest management strategy. However, further validation of causal relationships and elucidation of molecular mechanisms are required through experiments such as microbiota transplantation and targeted microbial depletion before such applications can be considered.

## Figures and Tables

**Table 1:** Scheirer-Ray-Hare for effects of photoperiod and temperature on α-diversity indices of gut microbiota in *L. sticticalis* larvae.

Parameters	Source	df	Sum of Squares	*H*	*p*
Shannon index	Photoperiod	1	36.12	0.4105	0.52171
	Temperature	3	262.50	2.9830	0.39426
	Photoperiod × Temperature	3	1361.38	15.4702	0.00146
	Residuals	24	1068.00		
Simpson index	Photoperiod	1	45.12	0.5128	0.47394
	Temperature	3	267.25	3.0369	0.38597
	Photoperiod × Temperature	3	1382.13	15.7060	0.00130
	Residuals	24	1033.50		
Chao1 index	Photoperiod	1	4.5	0.0512	0.82103
	Temperature	3	653.5	7.4316	0.05934
	Photoperiod × Temperature	3	860.0	9.7799	0.02053
	Residuals	24	1208.0		

**Table 2:** α-diversity indices of gut microbiota in *L. sticticalis* larvae under different photoperiod and temperature treatments.

Parameters	Temperature (°C)	L10:D14	L16:D8
Shannon index	18	4.80 ± 1.53 a	2.64 ± 1.33 ab
	22	4.98 ± 1.05 ab *	0.59 ± 0.30 b
	26	0.16 ± 0.01 b	3.21 ± 1.12 a *
	30	2.65 ± 0.59 ab	2.18 ± 0.62 ab
Simpson index	18	0.72 ± 0.23 ab	0.44 ± 0.18 a
	22	0.87 ± 0.08 a *	0.13 ± 0.07 a
	26	0.03 ± 0.00 b	0.66 ± 0.11 a *
	30	0.60 ± 0.10 ab	0.64 ± 0.03 a
Chao1 index	18	384.45 ± 120.34 a	253.40 ± 135.93 a
	22	265.84 ± 67.18 ab	63.48 ± 26.45 a
	26	11.88 ± 0.72 b	185.47 ± 68.12 a *
	30	136.67 ± 32.01 ab	99.94 ± 63.61 a

Different lowercase letters indicate significant differences among temperature treatments within the same photoperiod (*p* < 0.05, Kruskal–Wallis test with Dunn’s post-hoc test), and asterisks indicate significant differences between photoperiods at the same temperature (*p* < 0.05, Mann–Whitney test).

**Table 3:** Two-way PERMANOVA for effects of photoperiod, temperature, and their interaction on gut bacterial community structure of *L. sticticalis* larvae.

Source	df	Sum of Squares	*R* ^2^	F	*p*
Photoperiod	1	0.1029	0.0396	1.7101	0.171
Temperature	3	0.3361	0.1295	1.8622	0.113
Photoperiod × Temperature	3	0.7131	0.2747	3.9505	0.004
Residual	24	1.4440	0.5562		

PERMANOVA was performed on Bray–Curtis distances with 999 permutations.

## Data Availability

The raw 16S rRNA sequencing reads have been deposited in the Genome Sequence Archive (GSA) at the National Genomics Data Center (NGDC), China, under the accession number CRA046576 (BioProject: PRJCA068358). Processed data are included in the article and Supplementary Materials. Further inquiries can be directed to the corresponding authors.

## Supplementary Materials

The following supporting information can be downloaded at https://www.mdpi.com/article/10.3390/insects17080802/s1: Table S1: Results of high-throughput sequencing of 16S rRNA from gut bacteria of *L. sticticalis* under different temperature and photoperiod treatments; Table S2: Taxonomic composition of gut bacteria at various taxonomic levels in different temperature and photoperiod treatments of *L. sticticalis*; Table S3. Adonis pairwise comparisons of gut bacterial community structure among all treatment groups under different photoperiod and temperature combinations in *L. sticticalis*; Table S4. Relative abundances of the top 10 genera in the gut of *L. sticticalis* larvae under different photoperiod and temperature treatments; Figure S1. LDA score bar plot of LEfSe analysis for gut bacterial communities of *L. sticticalis* larvae under different treatments.

## Abbreviations

The following abbreviations are used in this manuscript:

L10:D14	Photoperiod of 10 h light:14 h dark
L16:D8	Photoperiod of 16 h light:8 h dark
G10.18	Treatment group with photoperiod L10:D14 and temperature 18 °C
G10.22	Treatment group with photoperiod L10:D14 and temperature 22 °C
G10.26	Treatment group with photoperiod L10:D14 and temperature 26 °C
G10.30	Treatment group with photoperiod L10:D14 and temperature 30 °C
G16.18	Treatment group with photoperiod L16:D8 and temperature 18 °C
G16.22	Treatment group with photoperiod L16:D8 and temperature 22 °C
G16.26	Treatment group with photoperiod L16:D8 and temperature 26 °C
G16.30	Treatment group with photoperiod L16:D8 and temperature 30 °C
CTAB	Cetyltrimethylammonium bromide
KEGG	Kyoto Encyclopedia of Genes and Genomes
ASV	Amplicon sequence variants
PC1	Principal coordinate 1
PC2	Principal coordinate 2
NMDS	Non-metric multidimensional scaling
