# Supplementary material for: Alterations in the Gut Microbiome of Loxostege sticticalis in Response to Temperature and Photoperiod Changes
Source: Insects. 2026 Aug 3;17(8):802. doi: 10.3390/insects17080802 (PMC13513058; doi:10.3390/insects17080802)
Supplement: Supplementary file 1 [file insects-17-00802-s001.zip › Supplementary Materials.pdf]

Alterations in the gut microbiome of *Loxostege sticticalis* in response to temperature and photoperiod changes

Jinghao Ji [1,2,†], Yanjun Xu [1,†], Yang Li [3], Hongxing Xu [2], Zhongxian Lu [2], Ronggen Wen [2,4], Sunan Shen [2,4], Yan Wu [4], Jianlei Huang [1,*], Jiawen Guo [2,*]

1 College of Agriculture and Forestry, Hebei North University, Zhangjiakou 075000, China; jijinghao2026@163.com (H. J.); XuYanJ@hebeinu.edu.cn (Y. X.); huangjl@hebeinu.edu.cn (J. H.)

2 State Key Laboratory for Quality and Safety of Agro-Products, Institute of Plant Protection and Microbiology, Zhejiang Academy of Agricultural Sciences, Hangzhou 310021, China; guojiawen91@126.com (J. G.); 13588332930@163.com (H. X.); luzxmh@163.com (Z. L.);

3 Lishui Municipal General Station of Soil, Fertilizer, Plant Protection and Energy, Lishui 323000, China; lishuizb@163.com (Y. L.)

4 Guizhou Provincial Key Laboratory for Rare Animal and Economic Insect of the Mountainous Region, Department of Biology and Engineering of Environment, Guiyang University, Guiyang 550005, China; rgw_ue@163.com (R. W.); s3234197322@163.com (S. S.); 15150535703@163.com (Y.W.)

* Correspondence: guojiawen91@126.com (J. G.); huangjl@hebeinu.edu.cn (J. H.). Tel.: +86-(0)571-88045127 (J. G.); +86-18931312951 (J. H.)

† These authors contributed equally to this work.

Table S1. Results of high-throughput sequencing of 16S rRNA from gut bacteria of *L. sticticalis* under different temperature and photoperiod treatments

Sample	Number of raw tags	Number of effective tags	Average length/bp	GC/%	Q20/%	Q30/%
G10.18.1	107566	104305	428.78	52.95%	98.53%	94.83%
G10.18.2	102434	85392	409.22	52.39%	98.48%	94.68%
G10.18.3	105021	94709	418.95	52.63%	98.28%	94.20%
G10.18.4	105717	91430	413.32	52.38%	98.46%	94.61%
G10.22.1	104984	89447	411.58	52.64%	98.42%	94.51%
G10.22.2	113889	87219	410.79	52.67%	98.51%	94.77%
G10.22.3	74116	63773	414.21	52.58%	98.19%	93.97%
G10.22.4	104731	100671	428.9	55.66%	98.47%	94.77%
G10.26.1	106805	104071	429	52.93%	98.66%	95.21%
G10.26.2	103327	98974	428.97	52.94%	98.27%	94.29%
G10.26.3	103219	100371	428.99	52.93%	98.62%	94.94%
G10.26.4	103974	101116	428.98	52.93%	98.71%	95.31%
G10.30.1	115040	107010	423.88	52.45%	98.43%	94.51%
G10.30.2	106111	102707	428.03	51.56%	98.45%	94.72%
G10.30.3	102980	99082	428.64	51.49%	98.37%	94.37%
G10.30.4	103732	94149	419.91	52.70%	98.40%	94.48%
G16.18.1	102297	87798	410.54	52.05%	98.43%	94.62%
G16.18.2	106725	102280	427.74	52.29%	98.61%	95.05%
G16.18.3	104612	100247	427.51	52.45%	98.45%	94.57%
G16.18.4	103026	97684	427.12	52.35%	98.51%	94.73%
G16.22.1	72946	70478	428.81	52.91%	98.56%	94.91%
G16.22.2	116816	113570	428.99	52.93%	98.50%	94.74%
G16.22.3	106705	103451	428.93	52.96%	98.35%	94.48%
G16.22.4	106740	103310	428.37	51.51%	98.46%	94.68%
G16.26.1	104748	101604	428.82	55.67%	98.59%	95.04%
G16.26.2	105127	101087	426.2	52.11%	98.54%	94.86%
G16.26.3	103798	93840	428.72	51.55%	98.10%	94.03%
G16.26.4	63660	56776	409.95	53.43%	98.66%	95.12%
G16.30.1	106034	102868	428.94	55.62%	98.71%	95.33%
G16.30.2	103408	100129	428.91	55.70%	98.60%	94.86%
G16.30.3	106639	103027	428.86	55.75%	98.67%	95.12%
G16.30.4	106416	92280	419.33	51.68%	98.54%	94.85%

Average length is the average length of valid tags. GC indicates the content of GC bases in valid tags. Q20 and Q30 are the percentages of bases in valid tags with base quality values greater than 20 (sequencing error rate less than 1%) and greater than 30 (sequencing error rate less than 0.1%).

Table S2. Taxonomic composition of gut bacteria at various taxonomic levels in different temperature and photoperiod treatments of *L. sticticalis*

Treatments	Number of ASVs	Phylum	Class	Order	Family	Genus	Species
G10.18.1	32	3	4	11	13	15	5
G10.18.2	559	11	16	37	57	73	40
G10.18.3	390	10	16	38	57	76	31
G10.18.4	504	16	19	44	61	72	31
G10.22.1	341	8	12	27	42	52	23
G10.22.2	366	13	16	34	55	65	33
G10.22.3	232	9	11	28	46	55	27
G10.22.4	79	7	9	22	29	38	11
G10.26.1	13	2	3	5	6	5	0
G10.26.2	11	2	2	2	2	2	0
G10.26.3	10	2	2	3	3	2	0
G10.26.4	10	2	2	3	3	3	0
G10.30.1	135	4	9	19	24	25	6
G10.30.2	90	7	8	17	23	22	4
G10.30.3	82	3	5	15	21	21	5
G10.30.4	221	7	9	25	38	49	15
G16.18.1	633	14	19	42	63	78	38
G16.18.2	106	5	9	17	25	24	7
G16.18.3	114	4	6	16	23	20	4
G16.18.4	126	8	12	26	34	37	17
G16.22.1	98	10	14	35	48	53	28
G16.22.2	13	2	2	4	4	4	0
G16.22.3	23	4	6	12	14	13	3
G16.22.4	112	5	7	19	30	29	5
G16.26.1	41	5	7	13	17	17	7
G16.26.2	261	16	24	51	75	100	36
G16.26.3	94	11	15	30	42	45	18
G16.26.4	331	10	18	43	78	93	51
G16.30.1	27	3	4	10	11	10	3
G16.30.2	33	4	6	12	17	18	3
G16.30.3	42	4	5	10	14	14	4
G16.30.4	281	8	11	30	52	62	22
Total	3645	29	52	128	205	407	281

Table S3. Adonis pairwise comparisons of gut bacterial community structure among all treatment groups under different photoperiod and temperature combinations in *L. sticticalis*

Group	Mean square	R^2	P value
G10.18-G10.22	0.226	0.110	0.514
G10.18-G10.26	1.030	0.526	0.031
G10.18-G10.30	0.865	0.367	0.071
G10.18-G16.18	0.823	0.358	0.065
G10.18-G16.22	0.724	0.302	0.078
G10.18-G16.26	0.555	0.209	0.163
G10.18-G16.30	1.033	0.384	0.06
G10.22-G10.26	1.543	0.630	0.026
G10.22-G10.30	0.929	0.387	0.053
G10.22-G16.18	0.895	0.381	0.065
G10.22-G16.22	1.122	0.405	0.063
G10.22-G16.26	0.467	0.184	0.468
G10.22-G16.30	0.690	0.297	0.062
G10.26-G10.30	1.557	0.734	0.029
G10.26-G16.18	1.580	0.743	0.036
G10.26-G16.22	0.127	0.146	0.206
G10.26-G16.26	1.404	0.545	0.022
G10.26-G16.30	1.628	0.690	0.032
G10.30-G16.18	1.045	0.485	0.04
G10.30-G16.22	1.012	0.436	0.026
G10.30-G16.26	0.196	0.102	0.664
G10.30-G16.30	0.992	0.434	0.048
G16.18-G16.22	1.171	0.476	0.066
G16.18-G16.26	0.850	0.331	0.057
G16.18-G16.30	1.219	0.488	0.035
G16.22-G16.26	0.919	0.324	0.077
G16.22-G16.30	1.140	0.436	0.038
G16.26-G16.30	0.501	0.208	0.215

Table S4. Relative abundances of the top 10 genera in the gut of *L. sticticalis* larvae under different photoperiod and temperature treatments.

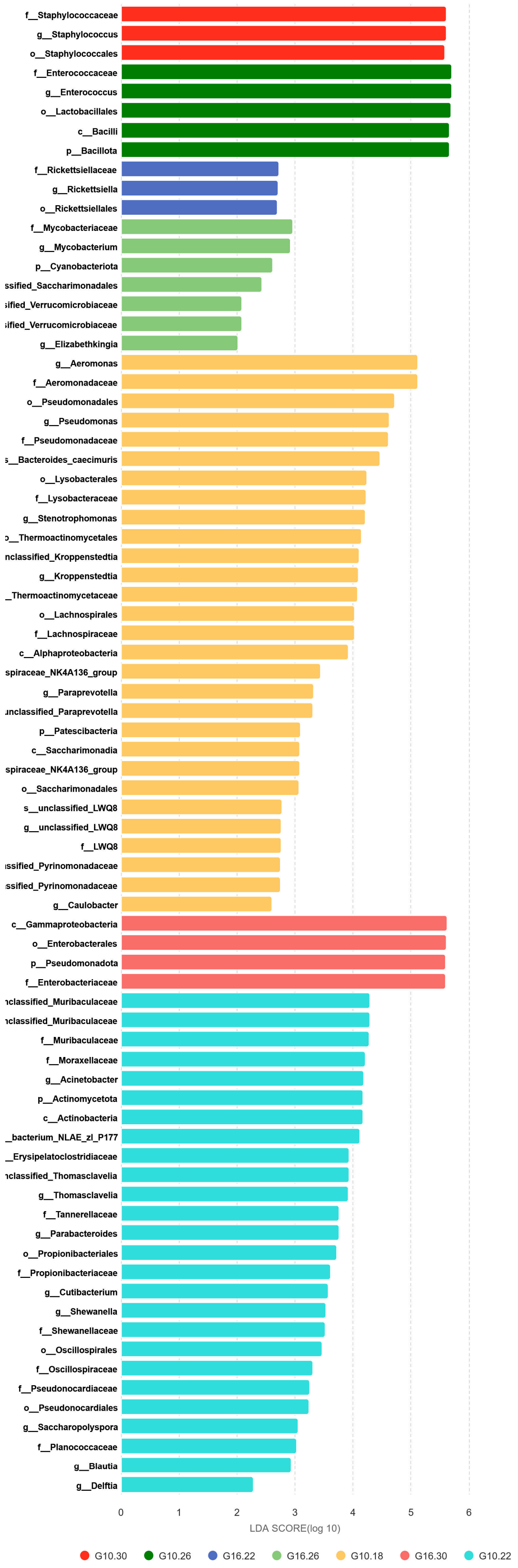

Figure S1. LDA score bar plot of LEfSe analysis for gut bacterial communities of *L. sticticalis* larvae under different treatments. LDA > 2.
